# Greater Occipital Nerve Block as an Effective Intervention for Medically Refractory Pediatric Migraine: A Retrospective Study

**DOI:** 10.7759/cureus.34930

**Published:** 2023-02-13

**Authors:** Refaat Hassan, Viral Gudiwala, Pretty Dawn, Louise Jeynes

**Affiliations:** 1 School of Clinical Medicine, University of Cambridge, Cambridge, GBR; 2 Pain Medicine, West Suffolk Hospital, Bury St Edmunds, GBR

**Keywords:** retrospective, headache, greater occipital nerve, pediatric migraine, nerve block

## Abstract

Background

Migraine is a common complaint worldwide, spanning all ages, but is not so well investigated in children and adolescents. Pediatric migraines incur significant health and social consequences with the most incisive effects seen in school performance, physical health, and mental well-being, making early effective management of pediatric migraine desirable. However, unlike adult migraine, the treatment for pediatric migraine has not been well established, which is especially true for the treatment of medically refractory migraine in children.

Methodology

We conducted a retrospective study to assess the feasibility of greater occipital nerve (GON) blocks as a therapeutic option for medically refractory migraine in children. We recruited subjects under 18 years old with a severe medically refractory headache that was affecting day-to-day life and who consented to receive a GON block. GON block effectivity was assessed through follow-up consultations and a post-procedural questionnaire.

Results

Six subjects received a first-time GON block for medically refractory migraine (mean age = 12 years and three months old, age range = 10 to 15 years old, three out of six subjects were female). We found that GON block was effective in all six patients for treatment of medically refractory migraine as assessed through follow-up consultations and a questionnaire sent out six weeks post-intervention. GON block was generally tolerable with only one patient reporting side effects (migraine flare-up for three days) post-intervention. Repeat injection was performed in four out of six patients, all of whom reported a repeat benefit.

Conclusions

We conclude that GON block is a feasible therapeutic option for the management of medically refractory migraine in children.

## Introduction

Migraine is a common complaint worldwide, but not so well investigated in children and adolescents as in adults. Migraine affects 8% of those 17 years old or younger with prevalence increasing with age, from 5% among children aged 5-10 years to 15% among adolescents [1]. Pre-pubescent males are more likely to suffer from migraine compared to pre-pubescent females, but by late puberty, migraine is more common in females [2]. Approximately 25% of children become migraine-free by the age of 25, and 50% by the age of 50, with remission being significantly more likely in males [3].

Differences in migraine symptomology between children and adults are well-characterized [4]. Compared to adults, school-aged children are more likely to report bilateral frontotemporal headaches of shorter duration, which are accompanied by abdominal pain or vomiting [3]. Migraines in infants younger than 12 months commonly manifest as episodes of headbanging while toddlers often look ill and express their pain through irritability [3]. Children may experience an aura prior to the onset of headaches, which is primarily noticed by changes in behavior in response to visual or auditory changes [5].

Pediatric migraines incur significant health and social consequences with the most incisive effects seen in school performance and physical health. Pediatric migraineurs reported reduced quality of life, increased absence from school activities, and poorer school performance compared to their migraine-free peers [6,7]. Additionally, more than one-third of pediatric migraineurs thought that their grades would improve without migraine [7]. Children with migraine did not participate in leisure activities for 24 days, on average, during the last three months because of migraine. Compared to non-migraineurs, pediatric migraineurs exercised less frequently and skipped meals more often [6,7]. Additionally, mood and anxiety symptoms are more likely to occur in children with migraine which can further reduce functioning and impair social interactions with peers, exacerbating effects on quality of life [8,9]. Undoubtedly, pediatric migraine confers significant challenges on parents and strains family interactions.

For all these reasons, early effective management of pediatric migraine is highly desirable. While there is a plethora of studies relating to the treatment of migraine in the adult population, due to difficulty in establishing studies in the pediatric population, there is a relative paucity of these, and, therefore, medical treatment of pediatric migraine is often extrapolated from adult data [10]. Which medication, if any, to use in treating pediatric migraine has not been established, with physicians facing considerable difficulty in finding effective and tolerable options in the pediatric population [11]. Furthermore, common treatments for adult migraine may not be as effective in children as well as potentially being a risk to the developing nervous system [11].

Nerve blocks are a well-established therapy for adult migraine where medical therapy has failed or is not tolerated, providing rapid and sustained relief from headaches [12]. Nerve blockade is achieved through the infiltration of the area with a local anesthetic, corticosteroid, or a combination of both [13,14]. In particular, the greater occipital nerve (GON) appears to be an ideal candidate given the convergence between spinal afferents providing sensory innervation from the C2 occipital region and trigeminal afferents at the trigeminocervical complex, a brain area implicated in primary headache disorders [14]. Corticosteroids reduce mechanical allodynia while local anesthetics decrease the sensation of afferent nerves in both the trigeminal and cervical nerves given this convergence within the GON [14,15].

Given the established effectivity of GON block in adults, we investigated the effectivity of GON block in pediatric migraines and the incidence of adverse effects.

## Materials and methods

Parental consent was provided for each GON block. Subjects under 18 years old with severe incapacitating migraine that was affecting day-to-day life and who found inadequate pain relief from conventional medical treatment including non-steroidal anti-inflammatory drugs and triptans were recruited from a single specialist pain center between June 2016 and February 2021. Patient consent to conduct the GON block was obtained for all included patients. Prior to GON block administration, a functional impact assessment was conducted for each patient which included school attendance, medication usage, mood, and engagement in pleasurable activities. Patients’ headache burdens were recorded as either mild, moderate, or severe during a pre-procedural review conducted by a pain specialist. Prior to GON block administration, a physical examination was conducted to screen for tenderness over the occipital region and locate the region of maximal tenderness.

The GON block consisted of 0.5% Chirocaine (levobupivacaine) and Depomedrone (methylprednisolone acetate), with exact doses depending on patients’ weight. The final solution was injected using a 25 G needle. A single specialist pain consultant performed all GON blocks after procedural consent was obtained. An alcohol-based swab was used to clean the occipital area prior to GON block administration. A landmark-guided approach was utilized, involving a single injection medial to the palpated occipital artery at the level of the superior nuchal line. Pressure was applied to the injected area afterward to minimize bruising.

GON block effectivity and possible adverse effects including local hair loss, bleeding, and migraine flare-up were assessed through questionnaires sent out via mail six weeks post-intervention (Table 1). The questionnaire focused primarily on three domains, namely, headache burden relief, improvement in quality of life, and satisfaction with intervention (Table 1). Recorded notes from follow-up consultations with the specialist pain consultant were also used to assess potential adverse effects and GON block effectivity.

**Table 1:** Six-week post-GON block questionnaire. GON = greater occipital nerve

Question	Answer options		
Did the intervention reduce your pain?	Yes, definitely	Yes, to some extent	No
If yes, how long did the effect last for?	>8 weeks	5–8 weeks	<4 weeks
What percentage of pain relief did you obtain when the intervention was working at its best?	>60%	30–60%	<30%
When the intervention was working at its best, did it improve sleep?	Yes, definitely	Yes, to some extent	No
When the intervention was working at its best, did it improve mobility?	Yes, definitely	Yes, to some extent	No
When the intervention was working at its best, did you manage to reduce pain medications?	Yes	N/A	No
If yes, by what percentage?	>50%	<50%	N/A
Did the intervention improve your quality of life?	Yes, definitely	Yes, to some extent	No
Is the intervention continuing to have an improvement on your quality of life?	Yes, definitely	Yes, to some extent	No
Would you have the procedure repeated?	Yes	No	N/A
Overall, how satisfied are you with the outcome of your intervention?	Excellent	Good	Fair
Have you experienced any side effects or complications?	No	Yes, to some extent	Yes, definitely

Data from questionnaire results and consultation notes were anonymized and extracted to Excel and subsequently analyzed.

## Results

Patient headache characteristics and medications prescribed are shown in Table 2. Patients’ ages (from date of birth to time of first GON block) ranged between 10 and 15 years old (mean = 12 years and three months old) and there was an even split of males and females. Exact GON block formulations for each patient are shown in Table 3.

**Table 2:** Patient characteristics.

Patient number	Age (years)	Sex	Type of headache	Headache burden	Comorbidities	Medications trialed	Examination findings
1	10	M	Migraine without aura with tension component, possible occipital neuralgia	Severe occurring daily. Described as “horrendous”	None	Paracetamol, ibuprofen, triptans	Trigger points over occipital nerves bilaterally. Tenderness over supraorbital nerves bilaterally
2	15	M	Migraine without aura with tension component, supraorbital nerve irritation	Severe weekly Fatigue. (wiped-out) for two days after the headache	None	Paracetamol, ibuprofen, triptans, topiramate	Trigger points over occipital nerves bilaterally. Tenderness over supraorbital nerves bilaterally
3	7	F	Migraine with aura	Severe, three times per week	Depression	Paracetamol, ibuprofen, triptans, topiramate	Tenderness over occipital nerves bilaterally
4	13	F	Migraine without aura	Severe, occurring every other day	None	Paracetamol, ibuprofen, triptans	Tenderness over occipital nerves bilaterally
5	14	M	Migraine, occipital nerve irritation	Severe, over 15 episodes per month	Chronic tic disorder, generalized anxiety disorder	Paracetamol, ibuprofen, triptans	Initially tender over the occipital nerve bilaterally with radiation superiorly. Tenderness has migrated to the supraorbital ridge over time
6	15	F	Migraine without aura	Severe, several times a week	Postural pre-syncope	Paracetamol, ibuprofen, propanol, triptans, topiramate	Tender over the occipital nerve bilaterally

**Table 3:** GON block formulation. GON = greater occipital nerve

Patient number	Block 1	Block 2 (months after the first block)	Block 3 (months after the second block)
1	1 mL 0.5% Chirocaine + 20 mg Depomedrone	N/A	N/A
2	2 mL 0.5% Chirocaine + 40 mg Depomedrone	1 mL 0.5% Chirocaine + 20 mg Depomedrone (seven months)	1 mL 0.5% Chirocaine + 10 mg Depomedrone (14 months)
3	1 mL 0.5% Chirocaine + 20 mg Depomedrone	N/A	N/A
4	1 mL 0.5% Chirocaine + 20 mg Depomedrone	N/A	N/A
5	1 mL 0.5% Chirocaine + 20 mg Depomedrone	1 mL 0.5% Chirocaine + 20.03 mg Depomedrone (10 months)	N/A
6	1 mL 0.5% Chirocaine + 40 mg Depomedrone	1 mL 0.5% Chirocaine + 20 mg Depomedrone (16 months)	1 mL 0.5% Chirocaine + 40 mg Depomedrone (eight months)

GON blocks were administered bilaterally in all six patients. Three patients received a single session of GON block, one patient received two separate sessions of GON block, and two patients received three sessions of GON block (Table 3). GON blocks were re-administered during follow-up appointments due to the diminishing therapeutic effects of the previous block.

Patient One (male 10 years old) reported long-lasting (over eight weeks) relief of headache intensity and frequency, from a baseline of severe, almost daily episodes following GON block. This reduction in headache symptomology was associated with improvement in mobility, sleep, and pain medication usage by over 50%.

Patient Two (female 15 years old) reported no migraines between their first and second GON block six months later and only one migraine in the year following her second GON block from a previous baseline of severe, weekly episodes. This reduction in migraine frequency was associated with improvements in mobility, sleep, and pain medication intake by over 50%.

Patient Three (male seven years old) complained of severe migraine with aura occurring thrice a week which was abolished for a short period (under four weeks) post-GON block. After this four-week period, he reported reduced headache severity with a concomitant reduction in pain relief medication by over 50%; however, headache frequency remained unchanged.

Patient Four (female 13 years old) reported a significant reduction in duration and intensity of migraine following GON block during a follow-up consultation, resulting in a discontinuation of prophylactic medication, although rescue medication was still occasionally being used.

Patient Five (male 14 years old) reported over 60% headache relief following his first GON block; however, this was not associated with a reduction in pain medication or subjective perception of quality of life improvement. The patient reported their second GON block 11 months later as ineffective; however, at follow-up three months after, the specialist pain consultant reported only slight tenderness over the occipital nerve point, where previously prior to GON block significant tenderness was noted bilaterally, radiating superiorly with pain now located over the supraorbital ridge. This supraorbital neuralgia was subsequently treated with a supraorbital nerve block and a lidocaine plaster over the supraorbital ridge when the effects of the blocks waned. A recent review, three years after the supraorbital nerve block, reported that his quality of life has improved with only occasional migraines and that he is not on any regular medication.

Patient Six (female 15 years old) reported long-lasting (over eight weeks) effective headache relief (>60%) following both GON blocks. The patient reported improvements in sleep, mobility, and an over 50% reduction in pain medication, noting that the intervention improved their quality of life. Migraine flare-ups occurred three days following their first block although there were no side effects following their second or third block, four months and one year after the first block, respectively.

## Discussion

We found that GON block was associated with relief of migraine burden in all six pediatric patients for treatment of medically refractory migraine. This improvement in headache pain was associated with improvement in sleep, mobility, and quality of life as well as a reduction in pain medication usage, diminishing the risk of medication overuse headaches. GON block was generally tolerable with only one patient reporting side effects (migraine flare-up for three days) post-intervention. There was no mention of adverse effects reported elsewhere in the literature such as injection-site hair loss, dizziness, or visual changes [16]. The treatment effect was typically prolonged with four out of five patients reporting symptom relief for over eight weeks; however, the treatment effect was variable in the efficacious population, ranging from under four weeks in one case to almost a year in another. Given the prolonged treatment effect size, a repeat GON block may be suitable to provide long-term relief from migraine with repeat courses.

Data on effect onset are generally unavailable; however, past studies have shown that headache relief occurs in a few days [17,18]. Given the rapid effect onset, besides its use as a long-term prophylactic treatment, a single GON block may be used as a bridging therapy while other prophylactic treatments are explored. Regarding Patient Five who reported their second GON block to be ineffective, given the change in tender points from the occipital region to the supraorbital ridge, it is possible that their etiology of headache may have changed over time to become less migrainoid to more tension-type in nature. In adults, occipital tenderness predicts a beneficial response from the GON block; hence, the lack of occipital tenderness in Patient Five may have predicted a treatment no-response for the second GON block [12,18]. The dissociation between reported headache relief and improvement in quality of life or medication reduction may also be explained by this shift in headache nature, given that quality of life and medication use improved after Patient Five’s subsequent supraorbital nerve block.

Limitations of this study include the small sample size, lack of a control group, and the inability to exclude a placebo effect. It is worth noting that this is an invasive procedure and the placebo response in the treatment of migraine is well-established [19]. A randomized double-blind controlled trial of GON injections in children with migraine is needed to assess whether the GON block is more effective than a placebo; however, there are considerable ethical constraints given that consent is required for a potential sham injection. While this study benefits from a single practitioner undertaking the nerve blocks, further exploration of the differing efficacies of drug combinations and doses would improve the understanding of pediatric GON blocks and may offer greater intervention efficacy.

## Conclusions

Chronic migraine is a debilitating condition in children with a dearth of well-established therapies, unlike in the adult population. We investigated the effects of greater occipital nerve blocks as a treatment for migraine refractory to standard medications. We found that GON block was well-tolerable and effective in all six subjects for headache burden relief and was associated with improvements in quality of life and concomitant reduction in pain medication usage.
